# The under investigated facet of the COVID-19 pandemic: Molecular analysis of secondary bacterial infections at a COVID dedicated intensive care unit within a tertiary care center in Lebanon

**DOI:** 10.3389/fmed.2023.1001476

**Published:** 2023-02-01

**Authors:** Ahmad Sleiman, Pascal Abdelkhalek, George Doumat, Frida Atallah, Lama Hamadeh, Pamela Moussa, Imad Bou Akl, Ghassan Dbaibo, George F. Araj, Souha S. Kanj, Rami Mahfouz, Ghassan M. Matar, Zeina A. Kanafani, Antoine G. Abou Fayad

**Affiliations:** ^1^Department of Experimental Pathology, Immunology and Microbiology, Faculty of Medicine, American University of Beirut, Beirut, Lebanon; ^2^Center for Infectious Diseases Research, American University of Beirut, Beirut, Lebanon; ^3^World Health Organization (WHO) Collaborating Center for Reference and Research on Bacterial Pathogens, Beirut, Lebanon; ^4^Division of Infectious Diseases, Department of Internal Medicine, American University of Beirut Medical Center, Beirut, Lebanon; ^5^Department of Pathology and Laboratory Medicine, American University of Beirut Medical Center, Beirut, Lebanon; ^6^Pillar Genomics Institute, American University of Beirut, Beirut, Lebanon; ^7^Pulmonary and Critical Care Division, Department of Internal Medicine, American University of Beirut Medical Center, Beirut, Lebanon

**Keywords:** COVID-19, secondary bacterial infections, carbapenem resistance, colistin resistance, NDM-5, NDM-7, *mcr-1.26*

## Abstract

**Background:**

The coronavirus disease 2019 (COVID-19) caused by the severe acute respiratory syndrome coronavirus 2 (SARS-CoV-2) has spread worldwide. Secondary bacterial infections are associated with unfavorable outcomes in respiratory viral infections. This study aimed at determining the prevalence of secondary bacterial infections in COVID-19 patients admitted at a tertiary medical center in Lebanon.

**Methodology:**

From May till November, 2020, a total of 26 Gram-negative isolates were recovered from 16 patients during the course of their COVID-19 infection with *Escherichia coli* being the most prevalent. The isolates were assessed for their antimicrobial susceptibility by broth microdilution against 19 antimicrobial agents from different classes. Whole genome sequencing of 13 isolates allowed the mining of antimicrobial resistance (AMR) determinants as well as mobile genetic elements and sequence types (ST). Finally, broth microdilution with three different efflux pump inhibitors [theobromine, conessine and PheArg-β-naphthylamide (PAβN)] was done.

**Results:**

Antimicrobial susceptibility testing showed that out of the 26 Gram-negative isolates, 1 (4%) was extensively drug resistant and 14 (54%) were multi-drug resistant (MDR). Whole genome sequencing results revealed a plethora of AMR determinants among the 13 sequenced isolates. Moreover, the 9 Enterobacterales and 4 *Pseudomonas aeruginosa* sequenced isolates belonged to 9 and 2 different ST, respectively. Using a variety of efflux pump inhibitors we demonstrated that only PAβN had a significant effect when combined with levofloxacin, and the latter regained its activity against two *P. aeruginosa* isolates.

**Conclusion:**

The identification of carbapenem and colistin resistant Gram-negative bacilli causing secondary bacterial infections in critical patients diagnosed with COVID-19 should be of high concern. Additionally, it is crucial to monitor and track AMR, post-COVID pandemic, in order to better understand the effect of this disease on AMR exacerbation.

## Introduction

A surprising cluster of pneumonia cases of unknown source arose in Wuhan, Hubei province, China in early December 2019. Some of the infected patients developed serious complications such as acute respiratory distress syndrome (ARDS) (1). January 2020, marked the identification of the disease as coronavirus disease 2019 (COVID-19) caused by a novel coronavirus, the severe acute respiratory syndrome coronavirus 2 (SARS-CoV-2) (2). In March 2020, after the drastic increase in the number of cases worldwide, the World Health Organization (WHO) declared COVID-19 as a global pandemic (3).

During the course of this pandemic, several studies were conducted to establish a diagnosis, treatment, and preventive measures for this disease. The general symptoms include fever, cough, apnea, and nausea, but the most serious complication that has been associated with COVID-19 was pneumonia. In addition to the respiratory system, this disease also affects the hepatic, gastrointestinal, and neurological systems (3).

The association of SARS-CoV-2 with other viruses, bacteria and fungi poses many challenges in diagnosing and treating patients with COVID-19 (4). Zhu et al. (5) found that bacterial coinfections were more prominent than viral or fungal coinfections. Influenza-associated bacterial coinfections were originally associated with a substantial increase in morbidity and mortality during pandemics (6). Even though, there is still no clear evidence on how the bacterial coinfection is affecting the outcome of the viral disease, the majority of the studies show that it can aggravate its outcome (7). In this context and the rise in antimicrobial resistance (AMR), it is necessary to conduct investigations regarding bacterial coinfections in COVID-19 patients. Therefore, this study aimed at determining the prevalence of secondary Gram-negative bacterial infections in COVID-19 patients admitted to the intensive care unit (ICU) at a tertiary medical center in Lebanon during a specific time period. This study also investigates the molecular mechanisms of AMR within these bacterial pathogens.

## Materials and methods

### Bacterial isolates

Between May and November 2020, 137 patients were admitted to the medical center as COVID-19 patients. A total of 37 patients were admitted to the ICU and 16 out of the 37 patients developed bacterial co-infections due to Gram-negative pathogens. During that period of time, no other co-infections were recorded. A total of 26 Gram-negative isolates were recovered from 16 patients during the course of their COVID-19 infection admitted to the ICU at a tertiary medical center in Lebanon. They were distributed as: 8 *Escherichia coli*, 6 *Pseudomonas aeruginosa*, 4 *Enterobacter cloacae*, 3 *Klebsiella pneumoniae*, 2 *Stenotrophomonas maltophilia*, 1 *Klebsiella oxytoca*, *Providencia stuartii*, and *Acinetobacter baumannii* Supplementary Table 1. These consecutive isolates were recovered as part of the routine bacteriology workflow in the Pathology and Laboratory Medicine Department at the American University of Beirut Medical Center.

### Antimicrobial susceptibility testing

Antimicrobial susceptibly testing was performed by broth microdilution against 19 different antimicrobial agents from different classes. Serial dilution was performed with concentrations ranging from 1,024 to 1 μg/ml and the plate was incubated at 37°C for 18–24 h. All the experiments were run in duplicates. The results were interpreted according to the CLSI M100 guidelines (8). Control strains *K. pneumoniae* ATCC^®^ 13883, *E. coli* ATCC^®^ 25922 and *P. aeruginosa* ATCC^®^ 27853 were used in parallel to monitor the minimal inhibitory concentrations (MIC) results.

### Whole genome sequencing

Based on the resistance profiles, a total of 13 Gram-negative isolates were selected for sequencing, distributed as: 6 *E. coli*, 4 *P. aeruginosa*, 2 *K. pneumoniae*, and 1 *E. cloacae*. These isolates were either MDR or extensively drug resistant (XDR). Isolates that fall outside these categories were not sequenced. To prepare whole-genome sequencing libraries, fresh cultures were grown on MacConkey agar and genomic DNA was extracted using QIAamp DNA Mini Kit (Qiagen, Valencia, CA, USA). Sequencing libraries were prepared using the Nextera XT library prep kit (Illumina GmbH, Munich, Germany) and sequenced on the Illumina MiSeq sequencer, 2 × 150 bp. Reads quality control and trimming was done using Trimmomatic (v.1.2.14) after which assembly of the genome was performed using Unicycler on Galaxy.^1^ Sequences were deposited in NCBI under the BioProject number PRJNA613441.

### Bioinformatic analysis

Antimicrobial resistance genes were acquired through ResFinder on Center of Genomic Epidemiology (CGE)^2^ and CARD.^3^ Plasmids harbored in each isolate were determined using PlasmidFinder on CGE.^4^ Sequence types (ST) were identified using MLST on CGE.^5^

### Efflux pump inhibitor assay

Following the analysis of the whole genome sequencing results, the presence of the *acrAB-TolC* and *MexAB-OprM* genes was investigated. The efflux pump inhibitor theobromine was used against *E. coli* isolates that harbored the *acrAB-TolC* gene and were resistant to ciprofloxacin and/or tetracycline. Moreover, the efflux pump inhibitors, conessine and PheArg-β-naphthylamide (PAβN), were used against *P. aeruginosa* isolates that harbored the *MexAB-OprM* and were resistant to levofloxacin. Their MIC were determined for both antibiotics with and without the selected efflux pump inhibitor. Antimicrobial/efflux pump inhibitor combinations experiment was performed by adding fixed concentrations of the inhibitor to the experimental wells of a standard broth microdilution assay. We followed CLSI guidelines in this assay. However, minor modifications to broth volumes were made in order to accommodate the presence of the efflux pump inhibitor while keeping the concentrations of the antimicrobials and the bacterial suspensions in accordance with CLSI recommendations. For the selected isolates, theobromine, conessine, and PAβN efflux pump inhibitors were used at a fixed concentration of 100 (9), 20, and 25 μg/ml (10), respectively. The MICs of the tested isolates were interpreted according to the CLSI M100 guideline (8).

### Data analysis

IBM SPSS version 20 was used to perform data analysis. Descriptive statistics for all variables was used to analyze the data. Fisher’s exact test and independent *t*-test were used to examine the association between baseline characteristics variables and mortality of COVID-19 patients after bacterial co-infection. The significance was set at *p* < 0.05.

### IRB approval

IRB approval for this work was awarded by the American University of Beirut Institutional Review Board under the IRB ID BIO-2020-0541.

## Results

### Baseline characteristics of COVID-19 patients after bacterial co-infection

Baseline characteristics are presented in Table 1. Sixty percentage of patients were males and the average age was 62.13 ± 21.05 years. All of the patients previously stayed in the ICU between 0 and 2 days within 30 days prior to admission to the COVID ICU. 14 (93.3%) patients had 0–2 days of mechanical ventilation use. 13 (87%) patients had 0–2 days prior hospital stay. Only 2 (13.3%) patients had diabetes, 3 (20%) patients had renal insufficiency, 3 (20%) patients had heart failure and 1 (6.7%) had malignancy. The baseline comorbidities are presented in Table 1.

**TABLE 1:** Baseline characteristics of post-COVID-19 patients after bacterial co-infection.

Characteristic (*n* = 15)	No (%)
Male gender	9 (60%)
Age in years (mean, SD)	62.1 (21%)
Diabetes	2 (13%)
Renal insufficiency	3 (20%)
Gastrointestinal disease	1 (7%)
Malignancy	5 (33%)
Albumin in mg/dl (mean, SD)	32.8 (5%)
**Hospital stay within 30 days**	
0–2 days	13 (87%)
3–5 days	2 (13%)
>6 days	0 (0%)
**Mechanical ventilation**	
0–2 days	14 (93%)
3–5 days	0 (0%)
>6 days	1 (7%)
Hemodialysis	1 (7%)
Heart failure	3 (20%)
Steroids past 30 days	1 (7%)
Admission duration in days (mean, SD)	44.5 (60%)
Time at risk in days (mean, SD)	13.1 (19%)

### The association between demographic characteristics and mortality rate

There was no statistically significant difference between baseline characteristics and mortality (Table 2).

**TABLE 2:** The association between demographic characteristics and mortality rate among COVID-19 patients after bacterial co-infection.

Characteristic (*n* = 15)	Outcome		
	Alive	Dead	*P*-value
Male gender	6 (66.7%)	3 (33.3%)	0.455
Age	58.3 ± 22.9	67.8 ± 18.3	0.278
Time at risk in days	14.6 ± 23.7	11.0 ± 9.9	0.367
Diabetes	2 (100%)	0	0.343
Renal insufficiency	2 (66.7%)	1 (33.3%)	0.659
GI diseases	1 (100%)	0	0.600
Malignancy	3 (60.0%)	2 (40.0%)	0.706
Albumin (mg/dl)	33.00 ± 3.92	32.50 ± 7.01	0.071
Hospital stay within 30 days			
0–2 days	9 (69.2%)	4 (30.8%)	0.143
3–5 days	0	2 (100%)	
**Mechanical ventilation**			
0–2 days	9 (64.3%)	5 (35.7%)	0.400
3–5 days	0	0	
> 6 days	0	1 (100%)	
Hemodialysis	1 (100%)	0	0.600
Heart failure	1 (33.3%)	2 (66.7%)	0.341
Steroids past 30 days	0	1 (100%)	0.400
Piperacillin/tazobactam use within 30 days of admission	2 (100%)	0	0.526
Carbapenems use within 30 days of admission	1 (25.0%)	3 (75.0%)	0.103
Fluoroquinolones use within 30 days of admission	2 (33.3%)	4 (66.7%)	0.131

### The association between complications and mortality

Patients who developed sepsis (*p* = 0.042) or septic shock (*p* = 0.044) had a statistically significant higher rate of mortality (60%). However, the results of the other complications showed no significant difference in outcomes.

### The association between therapy and mortality

11 (47.83%) patients were treated with combination therapy of antibiotics, out of which 6 (54.5%) were alive and 5 (45.5%) died. There was no statistically significant difference in outcomes between patients who took combination therapy and those who did not (*p* = 0.400). Furthermore, the average time to appropriate therapy was 0.95 ± 2.36 days for all the patients. The time to appropriate therapy for people who lived was 0.92 ± 2.14 days and for those who died was 1.00 ± 2.83 days. The difference between the two groups was not statistically significant (*p* = 0.994).

### Antimicrobial susceptibility testing

#### Enterobacterales

A total of 17 Enterobacterales isolates were included in this study, distributed as: 8 *E. coli*, 4 *E. cloacae*, 3 *K. pneumoniae*, 1 *K. oxytoca* and *P. stuartii*. Broth microdilution results showed that out of the 17 isolates, 11 (65%) were resistant to cefuroxime, tetracycline, and trimethoprim/sulfamethoxazole, 10 (59%) to aztreonam and fosfomycin, 9 (53%) to ceftolozane/tazobactam, 8 (47%) to ceftazidime, ciprofloxacin, levofloxacin, azithromycin, tigecycline, and piperacillin/tazobactam, 7 (41%) to cefepime, 5 (29%) to imipenem and ertapenem, 4 (24%) to meropenem, and colistin, and 3 (18%) to gentamicin. Moreover, all the isolates were susceptible to amikacin (Table 3 and Supplementary Tables 4, 5).

**TABLE 3:** Antimicrobial susceptibility testing results of the 17 Enterobacterales isolates.

	No. resistant (%)					
Antimicrobials	*E. coli* (*n* = 8)	*E. cloacae* (*n* = 4)	*K. pneumoniae* (*n* = 3)	*K. oxytoca* (*n* = 1)	*P. stuartii* (*n* = 1)	Total (*n* = 17)
Meropenem	3	1	0	0	0	4 (24%)
Imipenem	3	1	0	0	1	5 (29%)
Ertapenem	3	1	1	0	0	5 (29%)
Cefuroxime	7	1	3	0	0	11 (65%)
Ceftazidime	5	1	2	0	0	8 (47%)
Cefepime	5	1	1	0	0	7 (41%)
Aztreonam	7	1	2	0	0	10 (59%)
Ciprofloxacin	6	1	1	0	0	8 (47%)
Levofloxacin	6	1	1	0	0	8 (47%)
Colistin	1	1	1	0	1	4 (24%)
Amikacin	0	0	0	0	0	0 (0%)
Gentamicin	0	1	2	0	0	3 (18%)
Fosfomycin	2	3	3	1	1	10 (59%)
Azithromycin	5	1	1	0	1	8 (47%)
Tetracycline	6	1	3	0	1	11 (65%)
Tigecycline	2	2	3	0	1	8 (47%)
Trimethoprim/sulfamethoxazole	7	1	3	0	0	11 (65%)
Piperacillin/tazobactam	4	1	3	0	0	8 (47%)
Ceftolozane/tazobactam	5	1	3	0	0	9 (53%)

#### Pseudomonas aeruginosa

Broth microdilution results showed that out of the 6 *P. aeruginosa* isolates, 4 (67%) were resistant to meropenem, imipenem, and piperacillin/tazobactam, 3 (50%) to cefepime, ciprofloxacin, levofloxacin, and ceftolozane/tazobactam, 2 (33%) to aztreonam, and 1 (17%) to ceftazidime. Furthermore, all the isolates were susceptible to gentamicin, amikacin, and colistin (Table 4 and Supplementary Table 6).

**TABLE 4:** Antimicrobial susceptibility testing results of the 6 *P. aeruginosa* isolates.

Antibiotics	No. resistant (%)
Meropenem	4 (67%)
Imipenem	4 (67%)
Ceftazidime	1 (17%)
Cefepime	3 (50%)
Aztreonam	2 (33%)
Ciprofloxacin	3 (50%)
Levofloxacin	3 (50%)
Colistin	0%
Gentamicin	0%
Amikacin	0%
Piperacillin/tazobactam	4 (67%)
Ceftolozane/tazobactam	3 (50%)

#### Stenotrophomonas maltophilia

Broth microdilution results of the 2 *S. maltophilia* isolates showed that both isolates were resistant to trimethoprim/sulfamethoxazole. Moreover, both were susceptible to ceftazidime and levofloxacin (Supplementary Table 8).

#### Acinetobacter baumannii

Broth microdilution results showed that the *A. baumannii* isolate was susceptible to all the tested antimicrobial, including: meropenem, imipenem, ceftazidime, cefepime, ciprofloxacin, levofloxacin, colistin, amikacin, gentamicin, tetracycline, piperacillin/tazobactam, and trimethoprim/sulfamethoxazole (Supplementary Table 7).

### Whole genome sequencing

A total of 13 isolates were sequenced, distributed as: 6 *E. coli*, 4 *P. aeruginosa*, 2 *K. pneumoniae*, and 1 *E. cloacae*.

#### Enterobacterales

Whole genome sequencing results showed that the 9 Enterobacterales isolates belonged to 9 different ST. The 6 *E. coli* isolates belonged to ST69, ST131, ST167, ST617, ST624, and ST648. Moreover, the 2 *K. pneumoniae* isolates belonged to ST45 and ST307, and the *E. cloacae* isolate belonged to ST732.

A total of 134 AMR determinants were detected among the 9 sequenced Enterobacterales isolates. They encoded resistance to various antimicrobial families, such as: β-lactams, fluoroquinolones, aminoglycosides, and colistin. Several AMR determinants that encode resistance for β-lactams were detected, some examples include *bla*_CTX–M–15_ (*n* = 6), *bla*_TEM–1*B*_ (*n* = 6), *bla*_SHV–26_ (*n* = 1), *bla*_CMY–145_ (*n* = 1), *bla*_OXA–1_ (*n* = 5), *bla*_NDM–5_ (*n* = 3), and *bla*_NDM–7_ (*n* = 1). Moreover, those that encode resistance for aminoglycosides include *aph(3″)-Ib* (*n* = 7), *aac(6′)-Ib-cr* (*n* = 5), and *aadA* (*n* = 2). *FosA* (*n* = 3), *FosA3* (*n* = 2), and *FosA6* (*n* = 2) are some of the AMR determinants that encoded resistance against fosfomycin. Colistin resistance was due to the *mcr-1.26* gene in one *E. coli* isolates and mutation in the *parC* (*n* = 6) and *gyrA* (*n* = 6) in addition to other AMR determinants encoded resistance to fluoroquinolones. Resistance to disinfectants was encoded by *qacE* (*n* = 6), *sitABCD* (*n* = 4), *OqxA* (*n* = 2), and *OqxB* (*n* = 2) determinants (Supplementary Table 2).

A total of 27 plasmids were harbored among the 6 *E. coli* isolates, distributed as: IncFIB(AP001918) (*n* = 5), IncFIA (*n* = 4), IncFII (*n* = 4), Col(BS512) (*n* = 3), IncFIB(pB171) (*n* = 1), IncFIC(FII) (*n* = 1), IncI(Gamma) (*n* = 1), IncFIA (*n* = 1), IncN (*n* = 1), IncX4 (*n* = 1), IncQ1 (*n* = 1), Col(MG828) (*n* = 1), Col156 (*n* = 1), Col440I (*n* = 1), and p0111 (*n* = 1). Moreover, the 2 *K. pneumoniae* isolates harbored 3 plasmids, IncFIB(K) (*n* = 2), IncFII(K) (*n* = 2), and Col(pHAD28) (*n* = 1). The *E. cloacae* isolate harbored the IncHI2, IncHI2A, and IncX3 plasmids.

#### Pseudomonas aeruginosa

Whole genome sequencing results showed that the 4 *P. aeruginosa* isolates belonged to 2 different ST, distributed as: ST309 (*n* = 3) and ST111 (*n* = 1).

A total of 67 AMR determinants were detected among the 4 sequenced *P. aeruginosa* isolates. They encoded resistance to various antimicrobial families, such as: β-lactams, fluoroquinolones, and aminoglycosides. Resistance to β-lactams was encoded by several AMR determinants, including *bla*_OXA–50_ (*n* = 3), *bla*_OXA–395_ (*n* = 1), and *bla*_OXA–846_ (*n* = 3). *crpP* (*n* = 1) and mutation in the *gyrA* (*n* = 2) led to fluoroquinolones resistance. Fosfomycin resistance was encoded by *FosA* (*n* = 4) and that against aminoglycoside included *emrE* (*n* = 4), *aph(3′)-IIb* (*n* = 4), and *aac(6′)-29b* (*n* = 1) determinants. *Sul1* (*n* = 1) and *qacE* (*n* = 1) encoded resistance against sulphonamides and disinfectants, respectively, Supplementary Table 3.

### Efflux pumps inhibitors

#### Enterobacterales

Theobromine is an efflux pump inhibitor that inhibits the efflux of ciprofloxacin and tetracycline encoded by *acrAB-TolC* gene. Sequencing results showed that the latter was detected in 6 *E. coli* isolates out of the 9 sequenced Enterobacterales ones. Broth microdilution results, with and without theobromine, against ciprofloxacin and tetracycline showed that there was no change in the MIC of both antimicrobials in the presence of theobromine (Table 5).

**TABLE 5:** Broth microdilution results for ciprofloxacin and tetracycline, with and without theobromine, for 6 *E. coli* isolates.

	MIC (μg/ml) [Int]			
	Ciprofloxacin		Tetracycline	
Isolate code	Without theo	With theo	Without theo	With theo
*E. coli* 64	128 [R]	128 [R]	128 [R]	128 [R]
*E. coli* 65	64 [R]	64 [R]	256 [R]	256 [R]
*E. coli* 66	128 [R]	128 [R]	–	–
*E. coli* 67	16 [R]	16 [R]	256 [R]	256 [R]
*E. coli* 69	256 [R]	256 [R]	256 [R]	256 [R]
*E. coli* 71	–	–	64 [R]	64 [R]

Theo, theobromine; Int, interpretation; R, resistant.

#### Pseudomonas aeruginosa

Conessine and PAβN are efflux pump inhibitor that inhibits the efflux of levofloxacin encoded by *MexAB-OprM* gene in *P. aeruginosa*. Sequencing results showed that the latter was harbored in all the 4 sequenced *P. aeruginosa* isolates. However, this experiment was just done on the levofloxacin resistant (*n* = 3). Broth microdilution results, with and without conessine, showed that there was no change in the MIC of levofloxacin in the presence of conessine. However, the results in the presence and absence of PAβN showed a 16-fold decrease in the MIC of levofloxacin in 1 isolate, a 4-fold decrease in 1 isolate, and no change in MIC in 1 isolate (Table 6).

**TABLE 6:** Broth microdilution results for levofloxacin, with and without conessine and PheArg-β-naphthylamide, for 3 *P. aeruginosa* isolates.

	MIC (μg/ml) [Int]		
	Levofloxacin		
Isolate code	Without EPI	With conessine	With PAβN
PSA35	2 [I]	2 [I]	2 [I]
PSA52	64 [R]	64 [R]	16 [R]
PSA61	32 [R]	32 [R]	2 [I]

PAβN, pheArg-β-naphthylamide; EPI, efflux pump inhibitor; R, resistant; I, intermediate; Int, interpretation.

## Discussion

The mortality and morbidity rates in viral infections increase when it is coupled with secondary bacterial infections, especially in case of viral ARDS (11). SARS-CoV-2 can facilitate the attachment and colonization of bacteria to the respiratory tissues of the host. Likewise, secondary bacterial infections can facilitate the systemic spread of the virus, increasing the risk of systemic infections and sepsis (12). Around 200 million viral community acquired pneumonias occur every year and various studies addressed the issue of secondary bacterial infections and viral pneumonia (13). In the case of COVID-19, studies done in several countries showed a variation in the prevalence of bacterial secondary infections among patients diagnosed with the virus, ranging between 1 and 50% (12).

In this study, a total of 26 Gram-negative isolates were recovered from 16 patients in the course of their COVID-19 infection. *E. coli* was the most recovered bacterial pathogen, followed by *P. aeruginosa*, *E. cloacae*, *K. pneumoniae*, *S. maltophilia*, *K. oxytoca*, *P. stuartii*, and *A. baumannii*. Although the most frequently isolated bacteria causing secondary bacterial infection in COVID-19 patients varied from one study to another (12, 14–16). All obtained Gram-negative bacilli isolates from COVID-19 patients in our study, such as *E. coli* (14), *P. aeruginosa* (15), *K. pneumoniae*, *K. oxytoca* (12), *E. cloacae* (2), *A. baumannii* (16), and *S. maltophilia* (17) match those isolated in similar studies in the literature.

Broth microdilution results showed that out of the 17 Enterobacterales isolates, 1 *E. cloacae* was XDR, 11 (7 *E. coli*, 3 *K. pneumoniae*, and 1 *P. stuartii*) were MDR, 2 (1 *E. coli* and 1 *E. cloacae*) were resistant to 2 antimicrobials, 2 (1 *K. oxytoca* and 1 *E. cloacae*) to 1 antimicrobial, and 1 *E. cloacae* isolate was susceptible to all tested antimicrobials. Six (3 *E. coli*, 1 *K. pneumoniae*, 1 *E. cloacae*, and 1 *P. stuartii*) out of the 17 Enterobacterales isolates were carbapenem resistant. Falcone et al. also described secondary bacterial infection caused by carbapenem resistant Enterobacterales in COVID-19 patients (18). Moreover, 3 (1 *E. coli*, 1 *K. pneumoniae*; and 1 *E. cloacae*) out of the 17 Enterobacterales isolates were colistin resistant (*P. stuartii* was not included in the count). Out of 6 *P. aeruginosa* isolates, 3 (50%) were MDR, 2 (33%) were resistant to 2 antimicrobials each, and 1 (17%) was susceptible to all the tested antimicrobials. Carbapenem resistance was detected in 4 (67%) out of the 6 *P. aeruginosa* isolates. Gysin et al. similarly identified carbapenem resistant *P. aeruginosa* isolates causing secondary bacterial infection in COVID-19 patients (19).

Whole genome sequencing results showed that all the 9 Enterobacterales isolates belonged to different STs. The 6 *E. coli* isolates belonged to ST69, ST131, ST167, ST617, ST624, and ST648. Moreover, the 2 *K. pneumoniae* isolates belonged to ST45 and ST307, and the *E. cloacae* isolate belonged to ST732. Moreover, the 4 *P. aeruginosa* were distributed into 2 different STs: ST309 (*n* = 3) and ST111 (*n* = 1). During the time of the study, we did not observe a nosocomial outbreak in the COVID-19 dedicated ICU wing. The reason behind that might be the fact that the COVID critical care unit and the COVID unit of non-critically ill patients were newly established in March 2020 in preparation for the pandemic. Both units were physically placed in a separate building from the main hospital and were newly furbished with the necessary equipment. Medical teams covering these two units did not take care of any other patient population. All healthcare teams in the units were provided with personnel protective equipment and the training necessary for its use.

A plethora of AMR determinants were detected among the sequenced isolates. Carbapenem resistance gene *bla*_NDM–5_ was detected in 3 *E. coli* isolates belonging to ST167, ST617, and ST648. In china, an *E. coli* isolate belonging to ST167 and harboring the *bla*_NDM–5_ gene was recovered from a patient at the teaching hospital of Zhengzhou University. *E. coli* isolates belonging to ST167 are classified as an internationally disseminated clonal lineage and they are associated with the global spread of *bla*_CTX–M–15_ and *bla*_NDM_ genes in both humans and animals (20). The *E. coli* belonging to ST167 in our study harbored the *bla*_CTX–M–15_ as well as the *bla*_NDM–5_ gene. Tian et al. described an *E. coli* isolate belonging to ST617 harboring the *bla*_NDM–5_ gene isolated from a patient admitted to a Children’s Hospital in Shanghai, China (21). In 2019, an *E. coli* isolate recovered from a patient admitted to a Japanese university hospital was found to belong to ST648 and harbor the *bla*_NDM–5_ gene (22). Furthermore, in our study a *bla*_NDM–7_ gene was found to be harbored in an *E. cloacae* isolate belonging to ST732. In addition to that, an *E. coli* belonging to ST624 and harboring the *mcr-1.26* gene was detected in our study. Poirel et al. (23) reported 2 *E. coli* isolates belonging to ST624 and harboring the *mcr-1* gene obtained from 2 patients in South Africa. Three out of the 4 sequenced *P. aeruginosa* isolates harbored the *bla*_OXA–50_ gene and belonged to ST309. In Switzerland, sequencing results of 8 *P. aeruginosa* isolates recovered from COVID-19 patients showed that they harbor the *bla*_OXA–50_ gene (19). Taking into consideration what is already known regarding the accuracy of whole genome sequencing (WGS) and its benefits, the identification when compared between the latter method and the conventional microbiology were in complete agreement in this study. However, the superiority of WGS was displayed in the amount of information, the level of details and the time required for the experiment. Using conventional techniques would have required days in order to gather such information where with WGS this was possible in less than 48 h.

Disinfectants were extensively used as sanitizers against COVID-19 throughout the pandemic. However, this may lead to major consequences in terms of AMR. In our study, 4 AMR determinants (*OqxA*, *OqxB*, *qacE*, and *sitABCD*) that encode resistance against disinfectants were detected. *OqxAB* efflux pumps are encoded by two *OqxA* and *OqxB* genes that are localized in one operon. Plasmid-encoded *OqxA* and *OqxB* efflux pumps can confer resistance to several antimicrobial agents, such as fluoroquinolones, chloramphenicol, and tigecycline (24, 25). The *qacE* gene encodes the efflux of antiseptics, mainly quaternary ammonium compounds, and they can be found on plasmids (26). The function of the *sitABCD* gene is to mediate the transport of Mn^2+^ and a Fe^2+^ transporter and it can be harbored on plasmids (27). Since all of these genes can be plasmid-borne, there dissemination due to the irrational use of disinfectants may pose a great risk on public health.

The *acrAB-TolC* efflux pump gene was detected in 6 sequenced *E. coli* isolates. Theobromine was used as an inhibitor for this gene (28). The efflux pump inhibitor assay results showed that no change in the MIC of both ciprofloxacin and tetracycline occurred once theobromine was added. This shows that the *acrAB-TolC* efflux pump gene was not expressed and was not involved in the resistance against both antimicrobials in the 6 *E. coli* isolates. Moreover, both conessine and PAβN, were used as inhibitors for the *MexAB-OprM* efflux pump gene in the 3 sequenced *P. aeruginosa* isolates (28). Although the MIC of levofloxacin against the 3 *P. aeruginosa* isolates did not change after the addition of conessine. A 16-fold decrease in the MIC of levofloxacin in 1 isolate, a 4-fold decrease in 1 isolate, and no change in MIC in 1 isolate was detected once PAβN was added. This may show that conessine did not inhibit the activity of the *MexAB-OprM* efflux pump gene. However, the inhibition of the latter by PAβN led to a decrease in the MIC of levofloxacin in 2 *P. aeruginosa* isolates but none became susceptible. This may be due to the detected mutations in the *gyrA* determinants in both isolates.

## Conclusion

Further studies are required to investigate the association of comorbidities, complications during hospital stay, antimicrobial use and mortality in COVID-19 patients with secondary bacterial infections. Detection of carbapenem and colistin resistance in Gram-negative bacilli isolates causing bacterial secondary infection should be alarming. Accordingly, it is of high importance to monitor secondary bacterial infections in critical patients diagnosed with COVID-19. A limitation for this study was the inability to keep collecting more isolates and keep the surveillance active since the deadly wave in Lebanon began in December. A total lockdown took place in the country and hospitals became overwhelmed with dying patients. However, additional studies are needed to assess the impact of antimicrobial therapy on therapeutic outcome in COVID-19 patients to prevent antimicrobial overuse.

## Data availability statement

The datasets presented in this study can be found in online repositories. The names of the repository/repositories and accession number(s) can be found in this article/Supplementary material.

## Author contributions

AS, PA, LH, and PM contributed toward the execution of the microbiology experiments. GDo and FA collected the clinical data. GA supplied the clinical isolates. IB, GDb, SK, GA, RM, and GM contributed in writing the grant and the manuscript. AA led the microbiology experimental section. ZK led the clinical section. All authors contributed to writing and editing the final draft of the manuscript.

## Footnotes

1
https://usegalaxy.org

2
https://cge.food.dtu.dk/services/ResFinder/

3
https://card.mcmaster.ca/

4
https://cge.food.dtu.dk/services/PlasmidFinder/

5
https://cge.food.dtu.dk/services/MLST/
