# Supplementary material for: The under investigated facet of the COVID-19 pandemic: Molecular analysis of secondary bacterial infections at a COVID dedicated intensive care unit within a tertiary care center in Lebanon
Source: Front Med (Lausanne). 2023 Feb 1;10:1001476. doi: 10.3389/fmed.2023.1001476 (PMC9928946; doi:10.3389/fmed.2023.1001476)
Supplement: Supplementary file 1 [file Data_Sheet_1.PDF]

Supplementary Material

Supplementary data

Table S1: Microorganisms detected among COVID-19 patients.

**Microorganism**	**Site**
Acinetobacter baumannii	Urine
Enterobacter cloacae	Swab
Enterobacter cloacae	DTA
Escherichia coli	Sputum
Escherichia coli	Urine
Escherichia coli	Swab (abdominal wall abscess)
Escherichia coli	Blood
Escherichia coli	Urine
Escherichia coli	Urine
Escherichia coli	DTA
Escherichia coli	Sputum
Klebsiella pneumoniae	DTA
Klebsiella pneumoniae	Blood
Klebsiella pneumoniae	Sputum
Providencia stuartii	Urine
Pseudomonas aeruginosa	Blood
Pseudomonas aeruginosa	Urine
Pseudomonas aeruginosa	DTA
Pseudomonas aeruginosa	Biopsy Bone
Pseudomonas aeruginosa	DTA
Pseudomonas aeruginosa	Urine
Stenotrophomonas maltophilia	DTA
Stenotrophomonas maltophilia	DTA

Table S2: Antimicrobial resistance genes detected in the 9 sequenced Enterobacterales isolates.

AMR Family	AMR determinants	No. (%)
Beta-lactam	*bla*$_{CTX-M-3}$	2 (22%)
	bla$_{CTX-M-15}$	6 (67%)
	bla$_{TEM-1}$	1 (11%)
	bla$_{TEM-1B}$	6 (67%)
	bla$_{TEM-141}$	2 (22%)
	bla$_{TEM-206}$	2 (22%)
	bla$_{TEM-209}$	2 (22%)
	bla$_{TEM-214}$	2 (22%)
	bla$_{SHV-1}$	1 (11%)
	bla$_{SHV-26}$	1 (11%)
	bla$_{SHV-28}$	1 (11%)
	bla$_{SHV-78}$	1 (11%)
	bla$_{SHV-98}$	1 (11%)
	bla$_{SHV-106}$	1 (11%)
	bla$_{SHV-145}$	1 (11%)
	bla$_{SHV-179}$	1 (11%)
	bla$_{SHV-194}$	1 (11%)
	bla$_{SHV-199}$	1 (11%)
	bla$_{CMY-145}$	1 (11%)
	bla$_{OXA-1}$	5 (56%)
	bla$_{NDM-5}$	3 (33%)
	bla$_{NDM-7}$	1 (11%)
Colistin	*mcr-1.26*	1 (11%)
fluoroquinolone antibiotic	*emrB*	6 (67%)
	parC	6 (67%)

	emrA	6 (67%)
	gyrA	6 (67%)
	emrR	6 (67%)
	qnrS1	1 (11%)
	qnrB1	2 (22%)
	QnrB17	1 (11%)
aminoglycoside antibiotic	*aph(3')-Ia*	1 (11%)
	aph(3'')-Ib	7 (78%)
	aph(6)-Id	7 (78%)
	aac(3)-Iia	3 (33%)
	AAC(3)-Iie	3 (33%)
	aac(6')-Ib-cr	5 (56%)
	aadA	2(22%)
	aadA1	2 (22%)
	aadA2	3 (33%)
	aadA5	3 (33%)
	ANT(3'')	1 (11%)
	kdpE	6 (67%)
	acrD	6 (67%)
	mex	1 (11%)
fosfomycin	*PtsI*	1 (11%)
	GlpT	5 (56%)
	UhpT	4 (44%)
	mdtG	6 (67%)
	cyaA	3 (33%)
	FosA3	2 (22%)
	FosA	3 (33%)
	FosA6	2 (22%)
Nitroimidazole antibiotic	*msbA*	7 (78%)

Monobactam, cephalosporin	*MIR-11*	1 (11%)
Phenicol	*catB3*	5 (56%)
	catI	1 (11%)
	catA1	1 (11%)
	floR	1 (11%)
	catII	1 (11%)
	catA2	1 (11%)
elfamycin antibiotic	*EF-Tu*	9(100%)
Trimethoprim	*dfrA1*	1 (11%)
	dfrA7	1 (11%)
	dfrA12	4 (44%)
	dfrA14	4 (44%)
	dfrA17	3 (33%)
aminocoumarin antibiotic	*mdtC*	6 (67%)
	mdtB	6 (67%)
	mdtA	6 (67%)
Glycopeptide antibiotic	*BRP(MBL)*	3 (33%)
peptide antibiotic	*PmrF*	6 (67%)
	ugd	3 (33%)
	bacA	6 (67%)
	eptA	6 (67%)
	YojI	6 (67%)
	eptB	2 (22%)
	ArnT	2 (22%)
cephalosporin, penam	*ampC1*	5 (56%)
	ampC	5 (56%)
	ampH	6 (67%)
macrolide antibiotic	*SAT-2*	1 (11%)
tetracycline antibiotic	*tet(A)*	5 (56%)

	tet(B)	2 (22%)
	tetR	2 (22%)
	tet(D)	1 (11%)
	emrY	6 (67%)
	emrK	6 (67%)
sulfonamide antibiotic	*sul1*	7 (78%)
	sul2	8 (89%)
fluoroquinolone antibiotic, aminoglycoside antibiotic	*AAC(6')-Ib-cr5*	5 (56%)
aminoglycoside antibiotic, aminocoumarin antibiotic	*baeR*	7 (78%)
	baeS	6 (67%)
	cpxA	5 (56%)
macrolide antibiotic, fluoroquinolone antibiotic, penam	*mdtF*	6 (67%)
	mdtE	6 (67%)
	CRP	9 (100%)
	gadX	4 (44%)
	gadW	2 (22%)
MLS - Macrolide, Lincosamide and Streptogramin B	*mph(A)*	5 (56%)
	mdf(A)	6 (67%)
	emrE	4 (44%)
	erm(42)	1 (11%)
acridine dye, disinfecting agents and intercalating dyes	*qacEdelta1*	6 (67%)
fluoroquinolone antibiotic, cephalosporin, cephamycin, penam	*AcrE*	4 (44%)
	AcrF	6 (67%)
Carbapenem,peptide antibiotic, aminocoumarin antibiotic, rifamycin antibiotic	*LptD*	2 (22%)
Monobactam, carbapenem, cephalosporin, cephamycin, penam, penem	*OmpK37*	2 (22%)
	OmpA	2 (22%)
nucleoside antibiotic, acridine dye, disinfecting agents and intercalating dyes	*mdtN*	6 (67%)
	mdtH	6 (67%)
	mdtO	6 (67%)

	mdtP	6 (67%)
macrolide antibiotic, fluoroquinolone antibiotic, cephalosporin, cephamycin, penam, tetracycline antibiotic	*H-NS*	9 (100%)
	evgS	6 (67%)
	evgA	6 (67%)
Macrolide antibiotic, fluoroquinolone antibiotic, aminoglycoside antibiotic, carbapenem, cephalosporin, penam, peptide antibiotic, penem	*KpnG*	2(22%)
macrolide antibiotic, aminoglycoside antibiotic, cephalosporin, tetracycline antibiotic, peptide antibiotic, rifamycin antibiotic	*KpnF*	3 (33%)
	KpnE	3 (33%)
fluoroquinolone antibiotic, lincosamide antibiotic, nucleoside antibiotic, acridine dye, phenicol antibiotic, disinfecting agents and intercalating dyes	*mdtM*	5 (56%)
fluoroquinolone antibiotic, cephalosporin, glycylcycline, penam, tetracycline antibiotic, rifamycin antibiotic, phenicol antibiotic, triclosan	*Escherichia coli acrA*	7 (78%)
	acrR	6 (67%)
	soxR	6 (67%)
	marR	6 (67%)
	soxS	6 (67%)
	AcrS	5 (56%)
	acrB	6 (67%)
macrolide antibiotic, fluoroquinolone antibiotic, aminoglycoside antibiotic, carbapenem, cephalosporin, glycylcycline, cephamycin, penam, tetracycline antibiotic, peptide antibiotic, aminocoumarin antibiotic, rifamycin antibiotic, phenicol antibiotic, triclosan, penem	*marA*	6 (67%)
	TolC	6 (67%)
fluoroquinolone antibiotic, monobactam, carbapenem, cephalosporin, glycylcycline, cephamycin, penam, tetracycline antibiotic, rifamycin antibiotic, phenicol antibiotic, triclosan, penem	*ramA*	1 (11%)
Disinfectant	*sitABCD*	4 (44%)
	qacE	6 (67%)
	OqxB	2(22%)
	OqxA	2(22%)

Table S3: Antimicrobial resistance genes detected in the 4 sequenced *Pseudomonas aeruginosa* isolates.

AMR Family	AMR determinants	No. (%)
Beta-lactam	*bla*$_{OXA-50}$	3 (75%)
	bla$_{OXA-395}$	1 (25%)
	bla$_{OXA-846}$	3 (75%)
	bla$_{PAO}$	4 (100%)
Sulphonamide	*sul1*	1 (25%)
Aminoglycoside	*emrE*	4 (100%)
	aph(3')-IIb	4 (100%)
	aac(6')-29b	1 (25%)
Fluoroquinolone	*gyrA*	2 (50%)
	crpP	1 (25%)
Phenicol	*mexN*	4 (100%)
	mexM	4 (100%)
	catB7	4 (100%)
Triclosan	*OpmH*	4 (100%)
	TriC	4 (100%)
	TriA	4 (100%)
	TriB	4 (100%)
Peptide antibiotic	*cprS*	4 (100%)
	arnA	4 (100%)
	basR	4 (100%)
	basS	4 (100%)
	cprR	4 (100%)
Bicyclomycin	*bcr-1*	4 (100%)
Fosfomycin	*fosA*	4 (100%)
Fluoroquinolone antibiotic, aminoglycoside antibiotic, benzalkonium chloride	*PmpM*	4 (100%)
Fluoroquinolone antibiotic, diaminopyrimidine antibiotic, phenicol antibiotic	*rsmA*	4 (100%)
	MexE	4 (100%)

	MexT	4 (100%)
	OprN	4 (100%)
	MexF	4 (100%)
Monobactam, carbapenem, cephalosporin	*PDC-19a*	3 (75%)
Monobactam, carbapenem, cephalosporin, cephamycin, penam	*PDC-3*	1 (25%)
Macrolide antibiotic, tetracycline antibiotic, triclosan	*MexJ*	4 (100%)
	MexL	4 (100%)
	MexK	4 (100%)
Macrolide antibiotic, monobactam, tetracycline antibiotic, aminocoumarin antibiotic	*MuxA*	4 (100%)
	MuxB	4 (100%)
	OpmB	4 (100%)
	MuxC	4 (100%)
fluoroquinolone antibiotic, tetracycline antibiotic, acridine dye, disinfecting agents and intercalating dyes	*OpmD*	4 (100%)
	MexH	3 (75%)
	MexI	4 (100%)
	MexG	4 (100%)
Fluoroquinolone antibiotic, cephalosporin, glycylcycline, penam, tetracycline antibiotic, rifamycin antibiotic, phenicol antibiotic, triclosan	*YajC*	4 (100%)
Macrolide antibiotic, fluoroquinolone antibiotic, tetracycline antibiotic, acridine dye, phenicol antibiotic, disinfecting agents and intercalating dyes	*MexW*	4 (100%)
	MexV	4 (100%)
Macrolide antibiotic, fluoroquinolone antibiotic, cephalosporin, penam, tetracycline antibiotic, aminocoumarin antibiotic, diaminopyrimidine antibiotic, phenicol antibiotic	*Type A NfxB*	4 (100%)
Macrolide antibiotic, carbapenem, tetracycline antibiotic, acridine dye, diaminopyrimidine antibiotic, phenicol antibiotic, disinfecting agents and intercalating dyes	*mexP*	4 (100%)
	mexQ	4 (100%)
	opmE	4 (100%)
Macrolide antibiotic, fluoroquinolone antibiotic, aminoglycoside antibiotic, cephalosporin, penam, tetracycline antibiotic, aminocoumarin antibiotic, diaminopyrimidine antibiotic, phenicol antibiotic	*MexD*	4 (100%)
	OprJ	4 (100%)
	MexC	4 (100%)
Fluoroquinolone antibiotic, cephalosporin, glycylcycline, penam, tetracycline antibiotic, acridine dye, rifamycin antibiotic, phenicol antibiotic, triclosan, disinfecting agents and intercalating dyes	*soxR*	4 (100%)

Macrolide antibiotic, fluoroquinolone antibiotic, aminoglycoside antibiotic, carbapenem, cephalosporin, cephamycin, penam, tetracycline antibiotic, acridine dye, phenicol antibiotic, disinfecting agents and intercalating dyes	*MexZ*	4 (100%)
Macrolide antibiotic, fluoroquinolone antibiotic, monobactam, aminoglycoside antibiotic, carbapenem, cephalosporin, cephamycin, penam, tetracycline antibiotic, peptide antibiotic, aminocoumarin antibiotic, diaminopyrimidine antibiotic, sulfonamide antibiotic, phenicol antibiotic, penem	*MexB*	4 (100%)
	nalD	4 (100%)
	MexS	4 (100%)
	MexR	4 (100%)
	nalC	4 (100%)
	MexA	4 (100%)
	CpxR	4 (100%)
	ArmR	2 (50%)
Macrolide antibiotic, fluoroquinolone antibiotic, monobactam, aminoglycoside antibiotic, carbapenem, cephalosporin, cephamycin, penam, tetracycline antibiotic, peptide antibiotic, acridine dye, aminocoumarin antibiotic, diaminopyrimidine antibiotic, sulfonamide antibiotic, phenicol antibiotic, penem, disinfecting agents and intercalating dyes	*OprM*	4 (100%)
	ParS	4 (100%)
	ParR	4 (100%)
Disinfectant	*qacE*	1 (25%)

Table S4: Antimicrobial susceptibility results for all the *E.coli* isolates using broth microdilution.

Isolate	E. coli 64		E. coli 65		E. coli 66		E. coli 67		E. coli 68		E. coli 69		E. coli 70		E. coli 71	
Classification	ESBL-MDR		CRE-MDR		CRE-MDR		ESBL-MDR		MDR		CRE-MDR		Non-MDR		ESBL	
Antibiotics	MIC (μg/mL)		MIC (μg/mL)		MIC (μg/mL)		MIC (μg/mL)		MIC (μg/mL)		MIC (μg/mL)		MIC (μg/mL)		MIC (μg/mL)	
Meropenem (R≥ 4 μg/mL)	<1 S	S	128	R	128	R	<1	S	<1	S	32	R	<1	S	<1	S
Imipenem (R≥ 4 μg/mL)	<1	S	128	R	32	R	<1	S	<1	S	32	R	<1	S	<1	S
Ertapenem (R≥ 2 μg/mL)	<1	S	256	R	128	R	<1	S	<1	S	64	R	<1	S	<1	S
Cefuroxime (R≥ 32 μg/mL)	1024	R	>1024	R	>1024	R	>1024	R	1024	R	>1024	R	2	S	512	R
Ceftazidime (R≥ 16 μg/mL)	32	R	>1024	R	>1024	R	2	S	16	R	>1024	R	<1	S	1	S
Cefepime (R≥ 16 μg/mL)	128	R	>1024	R	>1024	R	8	S	128	R	512	R	<1	S	8	S
Gentamicin (R≥ 16 μg/mL)	<1	S	<1	S	4	S	<1	S	<1	S	2	S	2	S	<1	S
Ciprofloxacin (R ≥ 1 μg/mL)	128	R	64	R	128	R	16	R	<1	S	256	R	8	R	<1	S
Levofloxacin (R ≥ 2 μg/mL)	16	R	32	R	32	R	16	R	<1	S	32	R	8	R	<1	S
Colistin (R≥ 4 μg/mL)	<1	S	<1	S	<1	S	4	R	<1	S	<1	S	<1	S	<1	S
Amikacin (R≥ 64 μg/mL)	8	S	<1	S	8	S	2	S	2	S	8	S	2	S	<1	S
Tetracycline (R ≥ 16)	128	R	256	R	<1	S	256	R	<1	S	256	R	256	R	64	R
Tigecycline (R≥ 0.5 μg/mL)	<0.25	S	<0.25	S	<0.25	S	<0.25	S	<0.25	S	1	R	<0.25	S	0.5	R
Tazocin (R≥ 128/4 μg/mL)	2	S	>1024	R	>1024	R	4	S	<1	S	>1024	R	8	I	4	S
Zerbaxa (R ≥ 8/4 μg/mL)	4	R	>1024	R	>1024	R	<1	S	<1	S	>1024	R	<1	S	2	R
Fosfomycin (R ≥ 256 μg/mL)	8	S	4	S	16	S	>1024	R	8	S	16	S	4	S	>1024	R
Aztreonam (R ≥ 16 μg/mL)	64	R	8	I	>1024	R	8	I	128	R	>1024	R	<1	S	8	I
Azithromycin (R ≥ 32)	64	R	>128	R	128	R	64	R	2	S	>128	R	4	S	2	S
Bactrim (R ≥ 4/76 μg/mL)	>64	R	>64	R	>64	R	>64	R	>64	R	>64	R	1	S	>256	R

Table S5: Antimicrobial susceptibility results for all the remaining Enterobacterales isolates using broth microdilution.

Isolate	KLB5		KLB10		KLB11		KLB15		ENC10		ENC12		ENC13		ENC15		PDST16	
Classification	MDR		MDR		Non-MDR		MDR		XDR		Non-MDR		Non-MDR		Non-MDR		MDR	
Antibiotics	MIC (µg/mL)		MIC (µg/mL)		MIC (µg/mL)		MIC (µg/mL)		MIC (µg/mL)		MIC (µg/mL)		MIC (µg/mL)		MIC (µg/mL)		MIC (µg/mL)	
Meropenem (R≥ 4 µg/mL)	1	S	1	S	<1	S	<1	S	128	R	<1	S	<1	S	<1	S	<1	S
Imipenem (R≥ 4 µg/mL)	1	S	1	S	<1	S	<1	S	256	R	<1	S	<1	S	<1	S	2	I
Ertapenem (R≥ 2 µg/mL)	<1	S	4	R	<1	S	<1	S	128	R	<1	S	<1	S	<1	S	<1	S
Cefuroxime (R≥ 32 µg/mL)	256	R	>1024	R	<1	S	512	R	>1024	R	4	S	8	S	4	S	8	S
Ceftazidime (R≥ 16 µg/mL)	16	R	128	R	<1	S	2	S	>1024	R	<1	S	<1	S	<1	S	<1	S
Cefepime (R≥ 16 µg/mL)	8	S	512	R	<1	S	4	S	256	R	<1	S	<1	S	<1	S	<1	S
Gentamicin (R≥ 16 µg/mL)	64	R	128	R	<1	S	<1	S	32	R	<1	S	<1	S	<1	S	4	S
Ciprofloxacin (R ≥ 1 µg/mL)	32	R	<1	S	<1	S	<1	S	16	R	<1	S	<1	S	<1	S	<1	S
Levofloxacin (R ≥ 2 µg/mL)	8	R	<1	S	<1	S	<1	S	4	R	<1	S	<1	S	<1	S	<1	S
Colistin (R≥ 4 µg/mL)	<1	S	256	R	<1	S	<1	S	64	R	<1	S	<1	S	<1	S	>1024	R
Amikacin (R≥ 64 µg/mL)	2	S	<1	S	<1	S	<1	S	8	S	<1	S	<1	S	<1	S	4	S
Tetracycline (R ≥ 16)	256	R	512	R	<1	S	512	R	64	R	2	S	2	S	2	S	128	R
Tigecycline (R≥ 0.5 µg/mL)	2	R	1	R	<0.25	S	1	R	0.5	R	<0.25	S	<0.25	S	0.5	R	2	R
Tazocin (R≥ 128/4 µg/mL)	16	I	128	R	<1	S	16	I	>1024	R	4	S	2	S	2	S	<1	S
Zerbaxa (R ≥ 8/4 µg/mL)	4	R	64	R	<1	S	2	R	>1024	R	<1	S	<1	S	<1	S	<1	S
Fosfomycin (R ≥ 256 µg/mL)	256	R	256	R	256	R	512	R	128	I	512	R	32	S	128	I	128	I
Aztreonam (R ≥ 16 µg/mL)	32	R	512	R	<1	S	4	S	32	R	<1	S	<1	S	<1	S	>1	S
Azithromycin (R ≥ 32)	8	S	32	R	2	S	4	S	64	R	4	S	8	S	4	S	32	R
Bactrim (R ≥ 4/76 µg/mL)	>64	R	32	R	<0.25	S	>256	R	>64	R	<0.25	S	<0.25	S	0.5	S	2	S

Table S6: Antimicrobial susceptibility results for *P. aeruginosa* isolates using broth microdilution.

**Isolate**	**PSA32**		**PSA35**		**PSA45**		**PSA52**		**PSA61**		**PSA72**	
**Classification**	**Carbapenem Resistant**		**Carbapenem Resistant**		**Non-MDR**		**Carbapenem Resistant**		**Carbapenem Resistant**		**Non-MDR**	
**Antibiotics**	**MIC (µg/mL)**		**MIC (µg/mL)**		**MIC (µg/mL)**		**MIC (µg/mL)**		**MIC (µg/mL)**		**MIC (µg/mL)**	
Meropenem (R≥ 8 µg/mL)	16	R	32	R	<1	S	64	R	16	R	1	S
Imipenem (R≥ 8 µg/mL)	64	R	64	R	2	S	256	R	64	R	2	S
Ceftazidime (R≥ 32 µg/mL)	2	S	8	S	<1	S	16	I	8	S	2	S
Cefepime (R≥ 32 µg/mL)	2	S	16	I	<1	S	16	I	16	I	2	S
Gentamicin (R≥ 16 µg/mL)	<1	S	2	S	<1	S	2	S	4	S	<1	S
Ciprofloxacin (R ≥ 2 µg/mL)	<1	S	<1	S	<1	S	32	R	16	R	1	I
Levofloxacin (R ≥ 4 µg/mL)	<1	S	2	I	<1	S	64	R	32	R	1	S
Colistin (R≥ 4 µg/mL)	<1	S	<1	S	<1	S	<1	S	<1	S	<1	S
Amikacin (R≥ 64 µg/mL)	<1	S	4	S	<1	S	8	S	4	S	<1	S
Tazocin (R≥ 128/4 µg/mL)	4	S	16	I	4	S	16	I	16	I	8	I
Zerbaxa (R ≥ 16/4 µg/mL)	<1	S	2	I	<1	S	128	R	2	I	<1	S
Aztreonam (R ≥ 32 µg/mL)	8	S	32	R	4	S	16	I	8	S	2	S

Table S7: Antimicrobial susceptibility results for the *A. baumannii* isolate using broth microdilution.

**Isolate**	**ACN40**	
**Category**	**Non-MDR**	
**Antibiotics**	**MIC (µg/mL)**	
Meropenem (R ≥ 8)	<1	S
Imipenem (R ≥ 8)	<1	S
Ceftazidime (R ≥ 32)	4	S
Cefepime (R ≥ 32)	4	S
Gentamicin (R ≥ 16)	<1	S
Ciprofloxacin (R ≥ 4)	<1	S
Levofloxacin (R ≥ 8)	<1	S
Colistin (R ≥ 4)	<1	S
Amikacin (R ≥ 64)	<1	S
Tetracycline (R ≥ 16)	<1	S
Tazocin (R ≥ 128/4)	8	S
Bactrim (R ≥ 4/76)	<0.0625	S

Table S8: Antimicrobial susceptibility results for the *S. maltophilia* isolate using broth microdilution.

**Isolate**	STM9		STM14	
**Antibiotics**	**MIC (µg/mL)**		**MIC (µg/mL)**	
Ceftazidime (R ≥ 32)	<1	S	<1	S
Levofloxacin (R ≥ 8)	<1	S	<1	S
Bactrim (R ≥ 4/76)	32	R	32	R
